# Diagnostic Methods of* Helicobacter pylori* Infection for Epidemiological Studies: Critical Importance of Indirect Test Validation

**DOI:** 10.1155/2016/4819423

**Published:** 2016-01-19

**Authors:** Muhammad Miftahussurur, Yoshio Yamaoka

**Affiliations:** ^1^Department of Environmental and Preventive Medicine, Oita University Faculty of Medicine, Yufu 879-5593, Japan; ^2^Gastroentero-Hepatology Division, Department of Internal Medicine, Airlangga University, Faculty of Medicine, Surabaya 60131, Indonesia; ^3^Institute of Tropical Disease, Airlangga University, Surabaya 60115, Indonesia; ^4^Department of Gastroenterology and Hepatology, Baylor College of Medicine and Michael DeBakey Veterans Affairs Medical Center, Houston, TX 77030, USA

## Abstract

Among the methods developed to detect* H. pylori* infection, determining the gold standard remains debatable, especially for epidemiological studies. Due to the decreasing sensitivity of direct diagnostic tests (histopathology and/or immunohistochemistry [IHC], rapid urease test [RUT], and culture), several indirect tests, including antibody-based tests (serology and urine test), urea breath test (UBT), and stool antigen test (SAT) have been developed to diagnose* H. pylori* infection. Among the indirect tests, UBT and SAT became the best methods to determine active infection. While antibody-based tests, especially serology, are widely available and relatively sensitive, their specificity is low. Guidelines indicated that no single test can be considered as the gold standard for the diagnosis of* H. pylori* infection and that one should consider the method's advantages and disadvantages. Based on four epidemiological studies, culture and RUT present a sensitivity of 74.2–90.8% and 83.3–86.9% and a specificity of 97.7–98.8% and 95.1–97.2%, respectively, when using IHC as a gold standard. The sensitivity of serology is quite high, but that of the urine test was lower compared with that of the other methods. Thus, indirect test validation is important although some commercial kits propose universal cut-off values.

## 1. Introduction

*Helicobacter pylori* (*H. pylori*) infection is accepted as the primary cause of chronic gastritis [1]. Moreover, severe atrophic gastritis, accompanying intestinal metaplasia caused by persistent* H. pylori* infection, is closely related to the development of gastric cancer [2]. Although* H. pylori* was discovered more than 30 years ago by Marshall and Warren [3], which method should be considered as a gold standard for detection of* H. pylori* infection, especially for epidemiological studies, remains unclear. Currently, several direct diagnostic tests, including histopathology and/or immunohistochemistry (IHC), rapid urease test (RUT), and culture are frequently used as they provide genotype and antibiotic resistance information. However, due to the small amount of bacteria that colonizes the stomach, the direct test sensitivity decreases. Thus, several indirect tests, including antibody-based tests such as serology and urine test, urea breath test (UBT), and stool antigen test (SAT) have been developed to diagnose* H. pylori* infection [4].

Among the indirect tests, UBT is one of the most accurate to determine* H. pylori* infection with a sensitivity and specificity of 99% and 98%, respectively [5]. Together with SAT, UBT became the best method to identify active infection, which cannot be detected by serology [6]. However, some providers have modified a number of the UBT parameters, including the dose of isotope, duration of breath collection, requirement to fast, use of a test drink to slow gastric emptying, and analytical equipment [7]. Therefore, it is important to perform local validation. SAT is more economical than UBT and endoscopy to confirm the treatment success. However, differences in the antigens may affect the accuracy of this test in different populations. Although a meta-analysis revealed that SAT global sensitivity and specificity are more than 90% [8], a study reported low accuracy [9]. Moreover, by using new cut-off values after validation, the specificity increased by 20% [10]. On the other hand, antibody-based tests, especially serology, are widely available, inexpensive, not affected by local changes in the stomach, and suitable for special conditions. However, serological testing is less accurate than UBT and SAT, particularly in areas of low* H. pylori* prevalence [11, 12]. In a low-prevalence area, serological tests are not as effective. In a high prevalence area, a positive serology test result can reasonably be accepted as positive when there are no better alternative tests [7, 11]. The cut-off values should be validated locally although some commercial kits propose universal cut-off values.

We previously reported four epidemiological studies using four to five different tests to examine the prevalence of* H. pylori* infection in Dominican Republic [13], Bhutan [14], Myanmar [15], and Indonesia [16] (Table 1). To reduce a potential for bias, the same pathologist and microbiologist performed the experiments in all studies. We also used the same kits for culture and antibodies for IHC, RUT, and serology. When the study subjects were categorized as positive for* H. pylori* with at least one positive result, the overall* H. pylori* infection rate was 58.9%, 73.4%, 48.0%, and 11.5%, respectively. However, the sensitivity of the serology results in two studies, Bhutan [14] and Myanmar [15], was quite high compared with that of the other methods. In contrast, the sensitivity of the urine test used in the Indonesia [16] survey was lower than that of the other methods.

In this review, we highlighted the advantages and disadvantages of several methods used to diagnose* H. pylori,* including the importance of indirect test validation. We also summarized which methods are preferably recommended by several guidelines.

## 2. Direct Diagnostic Tests

Fiber optic endoscopy became very popular as it allows access to the stomach for the acquisition of biopsy specimens. Histology, RUT, and culture are methods used to detect* H. pylori* infection using biopsy specimens. On closer observation with standard endoscopy, especially in young patients,* H. pylori*-negative corporal mucosa presents star-fish like arrangements of vessels, termed “regular arrangement of collecting venules” with a sensitivity of 100% and a specificity of 90% [22, 23]. Narrow band imaging clearly shows superficial gastric mucosal and capillary patterns, indicating gastric mucosal abnormalities [24]. Moreover, using a novel ultra-high “magnified endoscopy” system (endocytoscopy), a moving bacterium can be visualized and recorded* ex vivo* at a 1100x magnification during endoscopy [25].

To minimize stomach invasiveness and overcome the lack of endoscopy equipment in our previous study [26], an extendable orogastric brush contained in a plastic tube (Baylor Brush, US Endoscopy, TX, USA) developed by Graham et al. [27] was used. The brush was about 5 mm in diameter and fitted within an enlarged distal sheath portion. Withdrawal of the brush into the sheath closed the brush compartment, allowing its extension and its movement back and forth 3–8 cm, three or four times. It was then immediately placed in a dram vial containing approximately 1 mL of cysteine transport medium with 20% glycerol [28]. This method appears to be reliable for the diagnosis of* H. pylori* infection in remote areas.

### 2.1. Histopathology and IHC

One advantage of this method is the possibility to send specimens via regular mail at room temperature, especially for epidemiological studies lacking freezing equipment. Our study in rural parts of Bhutan [14], Myanmar [15], and especially Indonesia [26] used histology confirmed with IHC as a gold standard to assess the sensitivity of the culture method (Table 1). Fixation with 10% formaldehyde provided very stable specimens, in which the morphology of the bacteria was maintained [29]. However, specimens should be stored for no more than one week [30].

For patients with gastric atrophy or intestinal metaplasia, histopathology presents a lower sensitivity [31]. A higher sensitivity was observed in the upper corpus gastric curvature, but not in antral biopsy for patients with gastric cancer [32]. In patients with extensive atrophy, a greater curvature of the corpus represents the optimal biopsy site, which presents a higher sensitivity than a lesser curvature of the corpus or the antrum (84.8%, 47.0%, and 30.3%, resp.) [33]. Therefore, for epidemiological studies, multiple biopsy specimens are necessary to increase the accuracy of this method. The updated Sydney system recommends that, for optimal assessment, biopsy specimens from five different sites should be obtained from the distal lesser and greater curvature of the antrum within 2-3 cm from the pylorus, two from the lesser and greater curvature of the corpus within 8 cm from the cardia, and one from the incisura angularis [34]. In our previous studies, the biopsy specimens were obtained following the Japanese guidelines [18], which recommend that biopsies should be performed on the greater curvature of the gastric antrum and in the upper to middle part of the gastric body, taking into account that* H. pylori* may be distributed unevenly in the stomach and that intestinal metaplasia can result in a false negative on the antral specimens [35, 36]. Comparison with a previous study showed an increase of about 10% in positivity [37]. In our survey, the detection rate of* H. pylori* infection using additional corporal biopsy specimens increased by 1–6% compared to that using antral biopsy specimens only (Table 2).

Several histochemical staining, including Warthin-Starry, Modified Giemsa, acridine orange, cresyl violet, Gimenez, Half Gram, Ziehl-Neelsen, Modified Genta, and* H. pylori* silver stain were used for the histological detection of* H. pylori* in gastric biopsies and could enhance the visualization of the organism compared to the routine hematoxylin and eosin (H&E) stain, which provided a weak contrast between bacteria and the mucus [29]. Although H&E sensitivity was comparable to that of Giemsa and Genta, the specificity decreased in low* H. pylori* density (90%) [38]. Warthin-Starry silver staining allows an excellent visualization, but is expensive, difficult to process, time consuming, and the results are not always reliable [39]. Modified Giemsa stain is feasible for* H. pylori* detection, being simple and presenting good contrast [29]. Several studies showed that IHC staining with specific* H. pylori* antibodies has the highest sensitivity and specificity and better interobserver agreement compared to histochemical stains [40]. It can also be used to assess the presence of* H. pylori* with more certainty, especially if there is evidence of inflammation and if coccoid forms of* H. pylori,* which mimic bacteria or cell debris and are difficult to identify by standard staining, are predominantly present as a result of hypoxia or other stress conditions [41, 42]. Moreover, IHC might be a useful tool for genotyping* H. pylori* without individual bias. Recently, we successfully generated an anti-East-Asian type CagA-specific antibody (*α*-EAS Ab), which was immunoreactivity with the East-Asian type CagA, but not with the Western type CagA [43]. We showed that *α*-EAS Ab was a useful tool for typing CagA immunohistochemically in Japanese [44], Vietnamese, and Thai [45] individuals with a sensitivity, specificity, and accuracy of 93.2%, 72.7%, and 91.6% and 96.7%, 97.9%, and 97.1%, respectively. Fluorescent* in situ* hybridization (FISH) is frequently used to detect* H. pylori* using 16S rRNA gene probe labeled with fluorescein [29]. A study investigated 201 gastric biopsy specimens comparing FISH with the conventional culture method. Although FISH is a more sensitive and rapid technique than the culture method for the detection of* H. pylori*, the combination of both FISH and conventional culturing significantly increased the sensitivity [46].

Building on current knowledge of the natural history of gastritis and the associated cancer risk, an international group of gastroenterologists and pathologists proposed a system for reporting gastritis in terms of stage, termed “Operative Link for Gastritis Assessment (OLGA)” [47]. Ninety-three Italian patients were followed up for more than 12 years. The data indicated that all invasive or intraepithelial gastric neoplasia were consistently associated with high-risk (III/IV) OLGA stages [48]. Our study showed that the distribution of OLGA score in these four countries tends to mirror the incidence rate of gastric adenocarcinoma (Table 3). Using OLGA score as a gold standard, we determined gastric mucosal atrophy and calculated the optimal cut-off points of pepsinogens in Myanmar and Bhutan [15, 17].

Several limitation of histology methods, including time and cost, dependence on the operator skills, and interobserver variability, should be considered [49]. Although an agreement was reached in the assessment of the density of* H. pylori*, inflammatory activity, chronic inflammation, and intestinal metaplasia by the Sydney classification updated in 1994, interobserver variability was common in biopsy specimens with lesser degrees of atrophy (weighted *K* value 0.49), particularly in the antrum [50]. Interpretation is especially difficult when tissue sampling is not adequate or if biopsies are not well-oriented. However, if hesitance occurs, the presence of active gastritis can be used as a surrogate pathognomonic of* H. pylori* infection. With regard to these limitations, we believed that histology using a valid staining is an excellent method and its accuracy can be increased by using IHC or FISH and with the acquisition of adequate multiple biopsy specimens.

### 2.2. Culture

Culture remains a reference method as it allows the direct detection of* H. pylori* organisms even though it presents a limited sensitivity and is a time-consuming procedure. It is highly specific and allows the determination of antimicrobial sensitivities. The sensitivity of the bacterium isolation varies greatly among laboratories due to a very fastidious organism. Even experienced laboratories recover the organism from only 50% to 70% of actually infected biopsies [51–53]. In our studies, the isolation sensitivity was between 74.2 and 90.8% when using histology confirmed by IHC as a gold standard method (Table 4). To increase sensitivity, care should be taken regarding the transport of biopsy specimens and storage, media plate, and microaerophilic conditions. Direct plating of biopsy samples may become the solution in areas where freezing equipment is not available, using disposable biopsy specimen grinders and microaerophilic gas generator packs. The transport medium is also essential for the successful detection of the bacteria. Saline solution was reported to be suitable for transport of less than four hours [29]. In our studies, we demonstrated that a cysteine transport medium containing 20% glycerol may be a good choice as we were able to recover 81% of the bacteria after one week of storage at 4°C [28].

To prevent the possible contamination by flora such as Gram-positive cocci from buccal or intestinal flora, in case of duodenal reflux, bacterial overgrowth, and* Candida* species from ulcers, several selective media such as Skirrow's, Dent's CP, modified Glupczynski's Brussels campylobacter charcoal media and chocolate agar medium were used for the isolation of* H. pylori.* These media contain antimicrobial compounds: vancomycin or teicoplanin, to inhibit Gram-positive cocci; polymyxin, nalidixic acid, colistin, trimethoprim, or cefsulodin to inhibit Gram-negative rods; and nystatin or amphotericin B to inhibit fungi [29]. Using a combination of two selective media was recommended for the maximum recovery of* H. pylori* [54]. Interestingly, although* H. pylori* colonizes the stomach and is sensitive to bile, which is present in the duodenum and colon, several studies succeeded in isolating* H. pylori* from stools [55–57]. As a fastidious bacterium, the massive number of microorganisms present in stools reduces the chances for* H. pylori* to grow. Special conditions such as pediatric (shorter intestinal transit time than adults), malnourished conditions (reduces gastric acid secretion), and fresh stool specimens (*H. pylori* may not survive for a long time in stools) may increase the success rate [58].

Recently, a novel fully automated rapid genetic analyzer was developed, which allows the determination of CAM resistance (e.g., 23S rRNA gene point mutations A2143G and A2144G) within 60–120 min without culture, while culture tests required 7–10 days [59]. This method may be useful in genotypic resistance-therapeutic guidance. However, using culture has other advantages. With PCR and/or next generation sequencing, we can screen mutations related to drug resistance. We previously discovered novel mutations related to clarithromycin resistance (infB and rpl22), which have synergic effects with 23S rRNA, resulting in higher minimum inhibitory concentrations (MICs) [60] using next generation sequencing. A new simple and rapid broth medium method was developed, which supports the growth of* H. pylori* for 20 hours and allows the bacterium detection using an enzyme-linked immunosorbent assay (ELISA) detection technique. When compared to the agar dilution method as a gold standard, 105 of 111 patients were detected as positive by both methods [61]. Moreover, clarithromycin and metronidazole susceptibilities were detected using this method, although 2 and 10 strains were misdiagnosed for clarithromycin and metronidazole susceptibility, respectively [61].

### 2.3. RUT

RUT presents the advantage of yielding results in 1–24 hours [12], making it a suitable method to detect* H. pylori* in epidemiological studies. In the presence of* H. pylori* urease, urea is hydrolyzed to produce ammonia and bicarbonate, leading to a pH increase in the gastric mucosa, which is indicated by a change in the color of phenol red from yellow to pink or red. After developing a medium to detect* H. pylori* with a pH indicator [62], McNulty et al. in 1989 performed a large trial on 1,445 patients undergoing upper gastrointestinal endoscopy over a 12-month period using two media, the original and modified Christensen's urea medium in which the concentration of phenol red is increased and the nutrients, glucose, and peptone are omitted [63]. Both media showed almost 100% specificity when compared with the culture method and histopathology [63]. The first-generation commercial kits were agar-based and were composed of campylobacter-like organism (CLO test; Kimberley-Clark, Neenah, WI, USA) containing antibacterial agents. Strip-based tests with two areas separated by a microporous membrane containing urea, a buffer, and a pH-sensitive indicator (PyloriTek, Serim, Elkhart, IN, USA) represent the new generation of commercial kits [29]. Another test used different indicators to start the reaction at lower pH in order to prevent contamination (false positive) from unrelated organisms (e.g., mouth flora) [64].

Beside treatment decision, RUT results could be used to predict the successful culture rate. Moreover,* H. pylori* could be successfully cultured from 84% and 100% of RUT positive samples [65], when CLO tests were kept at room temperature for 2 hours or at 4°C for 4 hours, respectively. Moreover, RUT samples can be used after 30 days of storage at room temperature for molecular testing to detect clarithromycin susceptibility [66]. However, although the color change usually occurs in less than 2 hours, it only become reliable after 4 hours when making a treatment decision [67]. Based on the literature, RUT samples should be discarded after 24 hours to avoid the detection of false positive from non-*H. pylori* urease containing organisms and should not be used to make treatment decision [64, 68]. In our experience, using CLO test, holding the samples for 24 hours is very important, especially for studies in low prevalence of* H. pylori* infection areas due to low colonization of* H. pylori*. However, we should consider that the main idea of RUT is to get rapid results for treatment decision. Recently, Vaira et al. designed a new RUT (UFT300, ABS Cernusco, Italy), which allows* H. pylori* detection within five minutes, with a sensitivity of 90.3, 94.5, and 96.2% at 1, 5, and 60 minutes, respectively (specificity was 100%) [69].

When using agar based test (CLO test), approximately 10^5^ of* H. pylori* bacteria are needed to induce a change in color, indicative of positivity [64]. We should consider that biopsy sample sites are very important based on the presence of this organism. Advanced gastritis and intestinal metaplasia will reduce the sensitivity of the test. Our epidemiological study showed that the sensitivity and specificity of RUT were 83.3–86.9% and 95.1–97.2%, respectively (Table 4). In this study, a single biopsy was taken from the antrum approximately 3 cm from the pyloric ring. Adding the number [70] and increasing the size [71] of biopsy specimens will increase the accuracy of RUT, especially if biopsies are obtained from the antrum and from the corpus, avoiding ulceration and intestinal metaplasia [64]. For bleeding patients and patients taking medications such as bismuth, antibiotics, or proton pump inhibitors (PPIs), the density and/or urease activity of* H. pylori* could be reduced and the test sensitivity could decrease to 25% [72]. Thus, patients should stop taking their medications two weeks before the diagnosis to prevent false negative. Formalin contamination of biopsy forceps may also generate false negative [73]. Several flora such as* Proteus mirabilis, Citrobacter freundii, Klebsiella pneumoniae, Enterobacter cloacae,* and* Staphylococcus aureus,* isolated from the oral cavity and/or stomach, also present urease activity [74] and can be potential false positive when using RUT.

## 3. Indirect Diagnosis

There are two types of indirect tests. Active tests, which detect active infection (UBT and SAT) and passive tests, which detect a marker of present/previous exposure to* H. pylori* (serology or urine), but do not indicate whether the infection is ongoing [75]. Although some of the tests present a high accuracy, the choice of the test to be used based on the clinical conditions should be determined taking into account local validation.

### 3.1. UBT

In a systematic review, Nocon et al. summarized 30 studies comparing the ^13^C-UBT to other tests. The ^13^C-UBT showed higher sensitivity and specificity than the IgG serology and SAT. However, the results were inconsistent when compared with RUT [76]. As mentioned above, this test cannot provide information about genotypes and antibiotic resistance. Moreover, it requires specialized equipment, which may not be available in routine clinical laboratories. Recently, a new portable ^14^C-based urea breath test (Heliprobe, Noster AB, Stockholm, Sweden) was produced, which is accurate, reliable, easy to use, fast (20 minutes), inexpensive, and uses low radioactivity of ^14^C-based urea capsule comparable to natural radiation [77].

The lower dose of ^13^C-UBT substrate (75–125 mg) was chosen with high accuracy compared to the original (350 mg) dose [78] and was validated in the United States [79] and Europe [80]. In general, UBT presents an excellent reliability when patients received pretreatment with citric acid and when the dose of ^13^C-urea administered is not lower than 75 mg to prevent poor results [81]. Compared to histology, urease test, and conventional UBT, a new UBT, consisting of two tablets each combining citric acid with 37.5 mg of ^13^C-urea, presents sensitivity and specificity >99% before and after treatment [82]. In contrast, Calvet et al. found an unexpectedly large number of false positive tests and an unacceptable low specificity (61%) when citric acid pretreatment was not included [83]. Citric acid could induce the rapid relaxation of the gastric fundus and a marked inhibition of the antral motility. Moreover, the simultaneous administration of substrate and a drink containing citric acid may significantly shorten the time required for the preparation of the test [84].

The progressive hypochlorhydria due to atrophy or use of acid-lowering medication could induce false-negative. The presence of atrophy, resulting in a lower load of bacteria, may produce false negative UBT. However, in combination with a serology test, UBT can be useful to diagnose* H. pylori* in patients with atrophic gastritis [85]. Some medications, including Bismuth containing compounds, antibiotics, and PPI, could decrease the test sensitivity through reduction of the organism density or urease activity. It is currently recommended that bismuth and antibiotics be withheld for at least 4 weeks and a PPI for 7–14 days prior to the UBT [12]. Udd et al. reported the importance of PPI discontinuation [86]. In fact, the utilization of high doses of PPI during 3 days leads to a negative UBT in 60% of the patients versus 27.5% for regular doses [86]. Moreover, Graham et al. also observed 33% negative UBTs after 6.5 days of PPI treatment and acidification of the stomach with citric acid did not improve the results [87]. On the other hand, false-positives may be due to contamination with non-*H. pylori* urease-producing bacteria [88, 89]. Sano et al. demonstrated that urease activity was also present in the oropharynx, therefore gurgling to eliminate urease-positive bacteria in the oropharynx and oral cavity is recommended [90].

The calculated optimal cut-off points of UBT expressed as delta over baseline (DOB) in a population in which a low prevalence of infection is expected (e.g., healthy volunteers) should be high. In contrast, a low DOB value should be observed in dyspeptic patients for whom the prevalence of infection is higher than in a normal population [84]. Using histology and microbiology, Mauro et al. calculated the cut-off point for the ^13^C UBT as 3.09%, 30 minutes after oral administration of 75 mg ^13^C-labeled urea in 100 mL of citric acid solution [91]. On the other hand, using a dose of 125-mg ^13^C urea and testing at 30 min, the accuracy was 94.8 with a cut-off point of 2.4% [79]. A multicenter Japanese study [92] defined the best cut-off value for children as 3.5%, 20 minutes after administration of 75–100 mg ^13^C urea with an overall sensitivity and specificity of 97.8% and 98.5%, respectively. Interestingly, DOB could also be used as a histological severity and eradication rate predictor. Pretreated patients with moderate to severe gastritis as assessed by histology presented higher DOB values compared to those with mild gastritis (34.5 ± 4.4 versus 17 ± 2.8), which was associated with a high* H. pylori* density [93]. High values of DOB (>35%) showed lower eradication rate (81.6% versus 94.7%) than a low DOB value (<35%) [94], and DOB values >15% could predict clarithromycin resistance [95].

### 3.2. SAT

In 1997, it was reported that the detection of* H. pylori* antigens in stools using polyclonal anti-*H. pylori* antibodies (HpSA) with a sensitivity and specificity of 88.8% and 94.5%, respectively [29]. However, due to the difficulty of obtaining polyclonal antibodies with constant quality, the tests using monoclonal antibody showed better accuracy. Gisbert and Pajares summarized 89 studies, including 10,858 untreated patients. The weighted mean sensitivity, specificity, positive predictive value, and negative predictive values were, 91%, 93%, 92%, and 87%, respectively. Even compared with UBT, the weighted mean sensitivity and specificity for SAT were 94% and 94%, respectively [96]. Between the two existing methods, enzyme immunoassay (EIA) presented a better accuracy than the immunochromatographic test, although the latter also used a monoclonal antibody [97, 98]. However, the immunochromatographic test is simple, user friendly, and does not require special equipment. Similar to UBT, the SAT sensitivity is affected by recent bismuth, antibiotics, and PPI treatments [12]. Fortunately, fasting is not needed for SAT and, recently, some monoclonal antibodies unaffected by PPI have been developed [99]. Therefore, SAT is more advantageous than UBT.

However, the submission of the stool sample is the main problem when using this test in epidemiological studies, especially in an area without freezing equipment. Stools should be stored at low temperature (−5 to −25°C) if not tested in short period of time (below seven days). Moreover, the samples should be stored at −80°C to maintain the antigen [98] for long time storage. Yee et al. reported that SAT still presented a good sensitivity and specificity, even with frozen stool samples stored (−80°C) for up to 225 days [100]. The conditions of the stool samples should be also taken into account. The accuracy of SAT decreases when the stool samples are unformed or watery due to diluted antigens [98]. The selection of the appropriate cut-off point represents a crucial factor, which is still debatable. Raguza et al. reported a high sensitivity, but low specificity of SAT using a monoclonal antibody (100% and 76.2%, resp.) when using the manufacturer's cut-off value. However when using a new cut-off (OD (1/4) 0.400), the sensitivity remained at 100%, but the specificity improved to 97.7% [10]. Therefore, a local test validation in order to find the best cut-off for each population may become very important.

### 3.3. Antibody-Based Tests

Serological tests that detect anti-*H. pylori* IgG antibodies could also lead to false-negatives. They are also less likely to be confounded by suppression of* H. pylori* infection by drugs for example, colloidal bismuth, PPI, or antibiotics [101]. Therefore, in particular clinical situations such as gastrointestinal bleeding [102], atrophic gastritis [103], gastric MALT lymphoma [104], and gastric carcinoma [105], serology is the most efficient diagnostic method [4]. However, this test cannot distinguish between current and past infections because* H. pylori* IgG persist even after the disappearance of this bacterium and returning to baseline values takes months or years although the bacterium eradication was successful [106]. False-negative results may occur for new infection when the antibody levels are not sufficiently elevated [29]. Interestingly, patients with atrophic corpus gastritis and with elevated* H. pylori* antibody titers, but ^13^C-UBT- and histology-negative for* H. pylori*, showed significantly decreasing titers in the eradication group compared with the follow-up subjects. Therefore, a positive serology result may indicate ongoing infection in spite of negative UBT and histology [103]. The standard ELISA and its derivatives such as rapid immunoenzymatic assays and immunoblotting are essential techniques with exact composition patient antigen [29].

**Figure 1:**
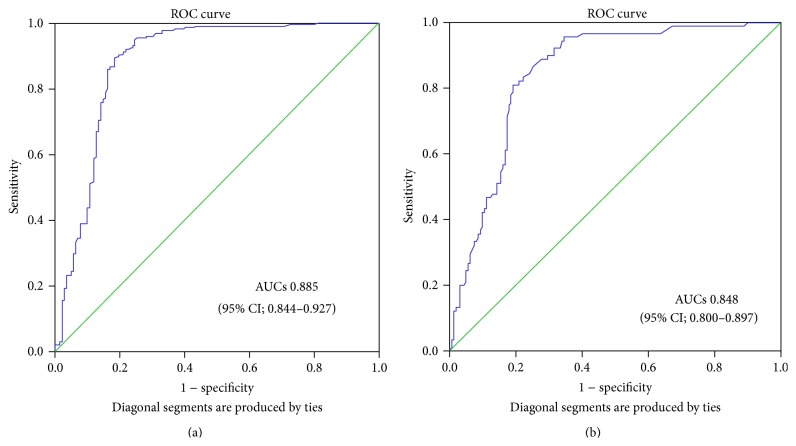
ROC curve for IgG ELISA in Bhutan (a) and Myanmar (b). The optimal cut-off points for the IgG ELISA were determined by analyzing the receiver-operating characteristic (ROC) using histology and immunohistochemistry as a gold standard.

Laheij et al. [107] reviewed 36 different commercially available* H. pylori* serology kits which had been used to screen 26,812 patients. Serology showed an excellent diagnostic performance when used in highly selected samples, but the performance decreased when tested in consecutive patient populations. The ranges of sensitivity and specificity were 57% to 100% and 31% to 100%, respectively, in different populations [107]. Another study evaluated 29 commercial kits, 15 of which were based on IgG ELISA. The sensitivity of ELISA ranged from 57.8% to 100%, and the specificity ranged from 57.4% to 97.9% [4]. Moreover, the diagnostic accuracy of kits made in Western countries has been reported to be lower in Chinese patients [108], and the imported serological kits yielded many intermediate results for Japanese patients. Therefore, their effectiveness seems somewhat limited in a Japanese patient population [109]. The difference of diagnostic performance depends on antibody preparation in every kit. Therefore, every serology tests must have been evaluated with indicated study population and the choice of the antigen is critical. In our studies, we quantified anti-*H. pylori* IgG levels using an ELISA kit (Eiken Co., Ltd., Tokyo, Japan), which was developed using Japanese* H. pylori* strains. In Bhutan, the serological test showed the highest positive rate (70.2%) compared with the other 3 tests (61.6%, 56.5%, and 54.6% for histology confirmed IHC, cultured, and RUT, resp.). When classifying* H. pylori*-positive with a* H. pylori* antibody titer ≥10 U/mL, the sensitivity and specificity were only 95.2% and 69.9% in Bhutan using histology confirmed by IHC as the gold standard. On the other hand, a low sensitivity (72.2%) was observed in Myanmar population (Table 4). Therefore, we calculated the best cut-off values of the IgG ELISA in Bhutan and Myanmar. By receiver operating characteristic curve (ROC), the best cut-off value of IgG ELISA was 13.5 in Bhutan (sensitivity and specificity were 90.4% and 80.3%, resp.), and the area under curve (AUC) was 0.885 (95% CI; 0.844–0.927) (Figure 1(a)). In contrast, the best cut-off value of IgG ELISA was 8.5 in Myanmar (sensitivity and specificity were 81.1% and 80.2%, resp.), and AUC was 0.848 (95% CI; 0.800–0.897) (Figure 1(b)).

Serological detection of the cytotoxin-associated gene product A (CagA) of* H. pylori* appears to correlate with further increases in risk for peptic ulcer disease and gastric cancer [110, 111]. Our meta-analysis showed that CagA seropositivity was higher in patients with gastric cancer than in controls, even in East-Asian countries with an overall OR of 1.26 (95% CI: 1.05–1.52) [112]. Asaka et al. reported that* H. pylori* antibody titer was significantly higher in patients with early gastric cancer than in advanced cancer [113]. The lower frequency of a higher IgG antibody titer in advanced cancer may be due to the increasing extent of intestinal metaplasia associated with the transition from the intestinal type of early gastric cancer to advanced cancer, such that the local environment is no longer ideal for* H. pylori* growth [113, 114]. CagA antibodies may be positive in patients who have a negative* H. pylori* serologic test [46, 47] since CagA antibodies can potentially remain positive for a longer period of time than the anti-*H. pylori* antibody [105, 115]. Therefore, a negative* H. pylori* serologic test does not rule out the possibility of a previous exposure to* H. pylori* and anti-CagA antibody alone is not a superior biomarker to the anti-*H. pylori* antibody alone.

A urine-based ELISA is an indirect, easy, rapid, and inexpensive test for the detection of an antibody to* H. pylori* in adults and has shown a high sensitivity and specificity [116–118]. However, in children, specificity was unacceptable (76.4%) and much lower than that for adults (91.5–100%) [118, 119]. The fact that* H. pylori* specific IgG are excreted in very low concentrations in urine may give rise to false negative results. The urine test presents several advantages and could become an alternative method for epidemiological and screening studies. Urine can be obtained easily and its collection requires little skills, does not require centrifugation, and is cheaper than that of serum [120]. In our studies, a rapid urine test (RAPIRUN* H. pylori* antibody, Otsuka Pharmaceutical Co., Tokyo, Japan), which has been reported to present a high accuracy, with excellent sensitivity and specificity, in Japanese (92.0%, 93.1%, and 92.3%, resp.) [121] and Vietnamese populations [122] was used. Although the results showed that the urine test was very specific, our study in Indonesia also showed a very low sensitivity (Table 4). It is possible that different genetic background of patients and* H. pylori* strains could induce different antigen-antibody responses that would affect the results of the urine test [120].

## 4. Diagnostic Methods Recommended by Several Guidelines

Table 5 provides a list of the available diagnostic tests for* H. pylori* recommended by several guidelines. Japanese guidelines recommend to simultaneously collecting biopsy specimens for histology when RUT is performed. If RUT is negative, histology examination is required for confirmation [18, 123]. American and Chinese guidelines recommend that biopsies for the RUT be obtained from two sites, the corpus at the gastric angularis and greater curvature of the antrum due to patchy distribution of* H. pylori* infection after antibiotics or PPIs [12, 19]. American guidelines recommend that RUT should rarely be used and should be combined with other endoscopic or nonendoscopic modalities [12]. In contrast, Chinese guidelines recommend RUT routine performance with high-quality testing reagents [19].

Five guidelines [6, 11, 12, 18, 19] mentioned culture as an optional method. However, they indicated that its sensitivity is lower than that of RUT or histology [12], the need for special transport [18], the demand for high techniques, the high cost, and availability in a limited number of clinical laboratories [12, 18], and it may not be practical in all countries [11].

All seven guidelines discussed the histology method. American guidelines [12] recommend that a minimum of three biopsies should be obtained, one from the angularis, one from the greater curvature of the corpus, and one from the greater curvature of the antrum, to maximize the diagnostic yield of histology. Japanese guidelines [18] also suggest the importance of IHC for distinguishing* H. pylori* from other microorganisms and for detecting coccoid forms of* H. pylori*. Chinese guidelines [19] also agree that IHC presents a high specificity, but a relatively high cost and that FISH has a high sensitivity in detecting* H. pylori* infection. Although PCR was mentioned by two guidelines [11, 12], it is not widely available for clinical use and not routinely recommended.

The UBT using essentially urea ^13^C [6, 7, 11, 12, 18, 19] or ^14^C [7, 11, 12, 19] remains accepted by the seven guidelines. The Asia-pacific consensus emphasized on the importance of local validation [7]. On the other hand, when UBT is positive, but the value of UBT is close to the cut-off value, the test could be resumed at a later period or* H. pylori* should be detected by using other methods [19].

SAT is accepted by seven guidelines, especially using monoclonal antibodies [6, 7, 18]. Despite being a good test, SAT may be underused due to its high costs in some countries/regions [11, 19].

Asia-pacific, European, and American guidelines recommend ELISA for IgG detection [6, 12], in addition to latex agglutination techniques or qualitative assessment using office-based kits [12]. Interestingly, while serology was not a recommended method for initial diagnosis of* H. pylori* infection in the absence of endoscopy by the Maastricht II consensus [124], the Maastricht III and IV consensus modified the guidelines stating that “some serological tests with good sensitivity and specificity can be used to perform the initial diagnosis of infection with* H. pylori*” [125] and only the validated commercial tests should be used [6]. Global and Japan guidelines also considered another source than serum: whole blood [11, 18], urine [18], and saliva [18]. All guidelines recommended the use of only validated commercial tests.* H. pylori* antibody kits with antigens extracted from domestic strains have been reported to be suitable for use in Japan, and the accuracy of testing for* H. pylori* antibody in urine samples is equal to or higher than that of serum testing [18]. Asia-pacific guidelines indicate that a high titer serological test is helpful to strengthen the diagnosis when histology is highly suggestive of infection. Serological testing may be helpful when the use of medication (PPI and antibiotic) cannot be avoided. Additionally, this method remains practical and reasonable for epidemiological studies [7, 19] and can be used as a diagnostic approach of current infection in patients with peptic ulcer bleeding or gastric MALT lymphoma [19].

Several guidelines indicate that not one single test can be considered the gold standard for the diagnosis of* H. pylori* [12] and that one should be chosen after considering the advantages and disadvantages of several tests [6, 11, 12, 18]. The Chinese consensus is that a current* H. pylori* infection can be diagnosed when one of the following three criteria is fulfilled: one of RUT, stained tissue section, and bacterial culture of gastric mucosal tissue is (1) positive; (2) positive ^13^C- or ^14^C-UBT; and (3) positive* H. pylori* stool antigen detection (by clinically verified monoclonal antibody). Republic of Korea guidelines indicate that the diagnosis* H. pylori* infection should include either one of the indirect methods (UBT, stool antigen test, or serum* H. pylori* IgG antibody test) or invasive methods (RUT or gastric biopsy for histology) [20].

## 5. Conclusions

Direct diagnostic methods, including histopathology and/or IHC, RUT, and culture are frequently used as they provide genotype and antibiotic resistance information. Among the indirect tests, UBT and SAT became the best methods to determine an active infection. On the other hand, antibody-based tests, especially serology, are widely available, very sensitive, but not specific. Based on four epidemiological studies, culture and RUT present a sensitivity of 74.2–90.8% and 83.3–86.9% and a specificity of 97.7–98.8% and 95.1–97.2%, respectively, when using histology and IHC as a gold standard. The sensitivity of the serology test is quite high, but that of the urine test was lower when compared with other methods. Several guidelines indicate that not one single test can be considered as the gold standard for the diagnosis of* H. pylori* and that the choice of the test should be made taking into consideration advantages and disadvantages of different methods (Table 6). Although there was no perfect test, the combination of culture confirmed by histology and IHC or combination of a validated serology and UBT will be complementary. The low sensitivity of culture will be complemented by histology, and IHC could increase the sensitivity of histology. On the other hand, serology will cover the weaknesses of UBT which has less ability in the presence of atrophy. However it should be noted that validation of indirect tests is important, although some commercial kits propose universal cut-off values.

## Figures and Tables

**Table 1:** Identification of *H*. *pylori* infection using different methods in four countries.

Country [ref.]	*n*	Histology + IHC	Culture	RUT	Other tests	At least one positive
Dominican Republic [13]	158	56.3%	43.0%	49.4%	—	58.9%
Bhutan [14]	372	61.6%	56.5%	54.6%	Serology 70.2%	73.4%
Myanmar [15]	252	35.7%	29.4%	34.1%	Serology 36.9%	48.0%
Indonesia [16]	78	7.7%	6.4%	9.0%	Urine test 5.1%	11.5%

IHC: immunohistochemistry; RUT: rapid urease test.

**Table 2:** Positivity of biopsy specimens by histology confirmed with IHC.

Country [ref.]	*n*	Both antrum and corpus-positive (%)	Antrum-positive (%)	Corpus-positive (%)	One positive of the two (%)
Dominican Republic [13]	158	82 (51.9%)	84 (53.2%)	87 (43.0%)	89 (56.3%)
Bhutan [14]	372	194 (52.2%)	206 (55.4%)	216 (56.5%)	229 (61.6%)
Myanmar [15]	252	72 (28.6%)	76 (30.2%)	86 (34.1%)	90 (35.7%)
Indonesia [16]	78	4 (5.1%)	4 (5.1%)	4 (5.1%)	6 (7.7%)

**Table 3:** Comparison of the OLGA system in Myanmar and Bhutan.

Country [ref.]	ASR for GC	Stage 0	Stage I	Stage II	Stage III	Stage IV
Dominican Republic [13]	8.3	22.1%	64.5%	13.2%	0.0%	0.0%
Myanmar [15]	15.3	43.2%	52.4%	4.0%	0.4%	0.0%
Bhutan [17]	23.0	7.8%	59.0%	27.5%	4.9%	0.8%
Indonesia [16]	3.9	65.3%	33.3%	1.2%	0.0%	0.0%

ASR: age-standardized incidence rate/100,000 (available from the International Agency for Research on Cancer; GLOBOCAN2012, http://globocan.iarc.fr/); GC: gastric cancer; OLGA: operative link for gastritis assessment.

**Table 4:** Accuracy of several tests using histology confirmed by IHC as a gold standard.

Type of tests	Sensitivity (%)				Specificity (%)			
	Dominican Republic [13]	Bhutan [14]	Myanmar [15]	Indonesia [16]	Dominican Republic [13]	Bhutan [14]	Myanmar [15]	Indonesia [16]
Culture	74.2	90.8	80.0	83.3	97.2	98.6	98.8	97.2
RUT	84.3	86.9	86.7	83.3	95.7	97.2	95.1	97.2
Serology	—	95.2	72.2	—	—	69.9	82.7	—
Urine test	—	—	—	50.0	—	—	—	98.6

IHC: immunohistochemistry; RUT: rapid urease test.

**Table 5:** Diagnostic methods of *H. pylori* infection recommended by several guidelines.

Guidelines	Invasive	Noninvasive
Global guidelines for developing countries [11]	Rapid urease test Histology Culture Fluorescence *in situ* hybridization polymerase chain reaction	Stool antigen test Finger-stick serology test Whole blood serology Urea breath test

Asia-Pacific consensus [7]	Rapid urease test Histology	Urea breath test Stool antigen test Serum antibody test (ELISA)

Europe [6]	Rapid urease test Histology Culture	Urea breath test Stool antigen test Serum antibody test (ELISA)

United States [12]	Rapid urease test Histology Culture Polymerase chain reaction	Urea breath test Stool antigen test Antibody test (quantitative and qualitative)

Japan [18]	Rapid urease test Histology Fluorescence *in situ* hybridization Culture	Urea breath test Antibody test (serum, whole blood, urine, and saliva) Stool antigen test

China [19]	Rapid urease test Culture Histology (Immunohistochemistry + fluorescence *in situ* hybridization)	Urea breath test Stool antigen test

Republic of Korea [20]	Rapid urease test Histology	Urea breath test Serum antibody test Stool antigen test

ELISA: enzyme-linked immunosorbent assay.

**Table 6:** Diagnostic test for *H. pylori* infection.

Diagnostic test	Sensitivity [18, 21]	Specificity [18, 21]	Advantages	Disadvantages
*Direct test*				
Histology	95%	99%	High accuracy, a possibility to send specimens at room temperature, and combination with IHC increase accuracy.	Low sensitivity for patients with gastric atrophy or intestinal metaplasia, time and cost, dependent on the operator skills, and interobserver variability.
Culture	69–98%	100%	Direct detection of *H. pylori*, excellent specificity, and allowing determination of antibiotic sensitivities.	Limited sensitivity, time-consuming procedure, and need of a special transport.
RUT	90%	93%	Inexpensive and provides rapid results, adding the number and increasing the size of biopsy specimens will increase the accuracy.	Sensitivity significantly reduced by bismuth, PPI and antibiotics, and formalin contamination of biopsy forceps generate false negative.

*Indirect test*				
UBT	95%	95%	Higher accuracy than serology and SAT, having a new portable type.	Atrophy, bismuth, PPI and antibiotics induce false-negative and need a local validation.
SAT	94%	92%	More economical than UBT and monoclonal antibody showed better accuracy.	Differences in the antigens may affect the accuracy, influence by bismuth, PPI, and antibiotics, and accuracy was influenced by stool condition.
Serology	90%	80%	Inexpensive, widely available, and the most efficient method in particular condition.	Less accurate than UBT and SAT and the cut-off values should be validated locally and cannot distinguish between current and past infections.

PPI: proton pump inhibitor; UBT: urea breath test; SAT: stool antigen test; RUT: rapid urease test.
